# Comparing the photocatalytic activity of TiO_2_ at macro- and microscopic scales

**DOI:** 10.1007/s11356-016-7887-3

**Published:** 2016-11-11

**Authors:** Antoni Torras-Rosell, Sabrina Rostgaard Johannsen, Kai Dirscherl, Svava Daviðsdóttir, Christian Sloth Jeppesen, Sascha Louring, Inge Hald Andersen

**Affiliations:** 1 DFM A/S, Danish National Metrology Institute, Matematiktorvet 307, Kongens Lyngby, DK-2800 Denmark; 2 Materials and Surface Engineering, Department of Mechanical Engineering, Technical University of Denmark, Produktionstorvet 425, Kongens Lyngby, DK-2800 Denmark; 3 Tribology Centre, Danish Technological Institute, DTI, Kongsvang Allé 29, Aarhus, DK-8000 Denmark

**Keywords:** TiO_2_, Photocatalytic activity, Kelvin probe force microscopy, Calibration, Thin films

## Abstract

This study focuses on the characterization of photocatalytic TiO_2_ coatings using Kelvin probe force microscopy. While most photocatalytic experiments are carried out at a macroscopic scale, Kelvin probe force microscopy is a microscopic technique that is surface sensitive. In order to link microscale results to macroscopic experiments, a simple method to establish the relation between Kelvin probe force microscopy and electrochemical measurements is presented by the calibration of a reference sample consisting of epitaxial deposited Cu-Ni-Au that is used as a transfer standard. The photocatalytic properties of TiO_2_ at macro- and microscopic scales are investigated by comparing photocatalytic degradation of acetone and electrochemical experiments to Kelvin probe force microscopy. The good agreement between the macro- and microscopic experiments suggests that Kelvin probe force microscopy can be a valuable tool towards the understanding, standardization and design of TiO_2_-based solutions in photocatalytic applications.

## Introduction

The benefits of nanomaterials and their contribution to economic growth and job creation have led the European Commission to identify nanotechnology as one of the key enabling technologies (KETs) that constitute a priority for European industrial policy (European Commission 2012a). However, a concern on safety aspects has also been raised in the European Commission due to the limited amount of data on manufactured nanoparticles and the major technical challenges on the characterization and analysis of nanoparticles (European Commission 2012b). In this respect, SETNanoMetro is a European project within the Seventh Framework Programme (FP7) that aims at determining the properties of titanium dioxide (TiO_2_) nanoparticles and thin films with various measurement techniques, and establishing a metrological chain of traceability to ensure the reliability of the results. One of the studied properties in SETNanoMetro is the photocatalytic activity of TiO_2_, which is activated by ultraviolet (UV) light. The photocatalytic activation results in the creation of reactive species on the surface of TiO_2_. These reactive species have a strong decomposing power which result in TiO_2_ having both self-cleaning and superhydrophilic properties. There exists different polymorphs of TiO_2_, where the anatase and rutile phases show the best potential for photocatalytic applications (Scanlon et al. 2013; Luttrell et al. 2014). Different types of experiments are employed to characterize the photocatalytic activity of TiO_2_, e.g., by measuring the photovoltage/photocurrent generated in an electrochemical experiment (Daviðsdóttir et al. 2014) or by looking at the degradation rate of methylene blue (Mills and Wang 1999; Ohko et al. 2001; Kotani et al. 2001; Fujishima et al. 2008). The photocatalytic activity is normally measured at a macroscopic scale, while any crystallographic study must at least be conducted at a microscopic scale. Despite numerous investigations (Linsebigler et al. 1995; Fujishima et al. 2008; Scanlon et al. 2013; Luttrell et al. 2014), a thorough knowledge of the correlation between the crystallographic structure and the electrical properties of TiO_2_ nanoparticles or thin films have not been established yet. Sample preparation is crucial for the photocatalytic performance. For instance, studies have demonstrated that the photocatalytic behavior of TiO_2_ coatings can vary substantially depending on their thickness (Daviðsdóttir et al. 2013) or the used metal substrate as well as the coating-substrate interface structure (Daviðsdóttir et al. 2013; Daviðsdóttir et al. 2014). This knowledge is therefore essential for optimizing manufacturing processes of TiO_2_-based solutions, which are nowadays commonly adjusted on a trial and error basis.

In the recent years, a number of studies have focused on the synthesis of TiO_2_ with very well-defined crystallographic orientations, and the assessment of the photocatalytic performance of such structures by means of macroscopic techniques (Hotsenpiller et al. 1998; Lowekamp et al. 1998; Ohno et al. 2002; Pan et al. 2011; Ahmed et al. 2011). No doubt that the photocatalytic activity is to be ultimately effective at a macroscopic level for most practical applications, but from a perspective of designing new types of nanoparticles, and for establishing traceability, it is desirable to observe the photocatalytic capabilities of tailor-made TiO_2_ nanoparticles already in the microscale domain. Consequently, in this paper, we present a study of the electrical changes on the surface of TiO_2_ at the microscopic level, when it is exposed to UV light. This is achieved by means of Kelvin probe force microscopy (KPFM), a microscale technique that is surface sensitive (Nonnenmacher et al. 1991; Weaver and Abraham 1991; Nabhan et al. 1997; Kitamura and Iwatsuki 1998; Hiehata et al. 2007). The present work demonstrates that KPFM is suitable for the characterization of photocatalytic TiO_2_, as photocatalysis occurs at the surface of TiO_2_, and focuses on comparing homogeneous TiO_2_ thin films on the micro- and macroscale. In the future, KPFM can potentially be used to determine the photocatalytic activity of single nanoparticles due to the microscopic nature of the technique.

The first part of this paper (“3Kelvin probe force 3microscopy”) presents KPFM as a microscopic technique, and then focuses on establishing a simple calibration procedure between surface potential measurements in KPFM and macroscopic electrochemical measurements based on open circuit potential (OCP). This is achieved by means of a reference sample consisting of three metal layers, where the potential difference among the three materials is determined by both techniques. In the second part (“3Assessing photocatalytic activity with 3KPFM” and “3Linking photocatalytic properties at both 3macro- and microscopic scales”), the principles for assessing the photocatalytic activity of TiO_2_ using KPFM are explained. The correlation between micro- and macroscopic properties is illustrated by comparing KPFM measurements to macroscopic results obtained via electrochemical experiments and assessment of acetone degradation.

## Kelvin probe force microscopy

**Fig. 1:**
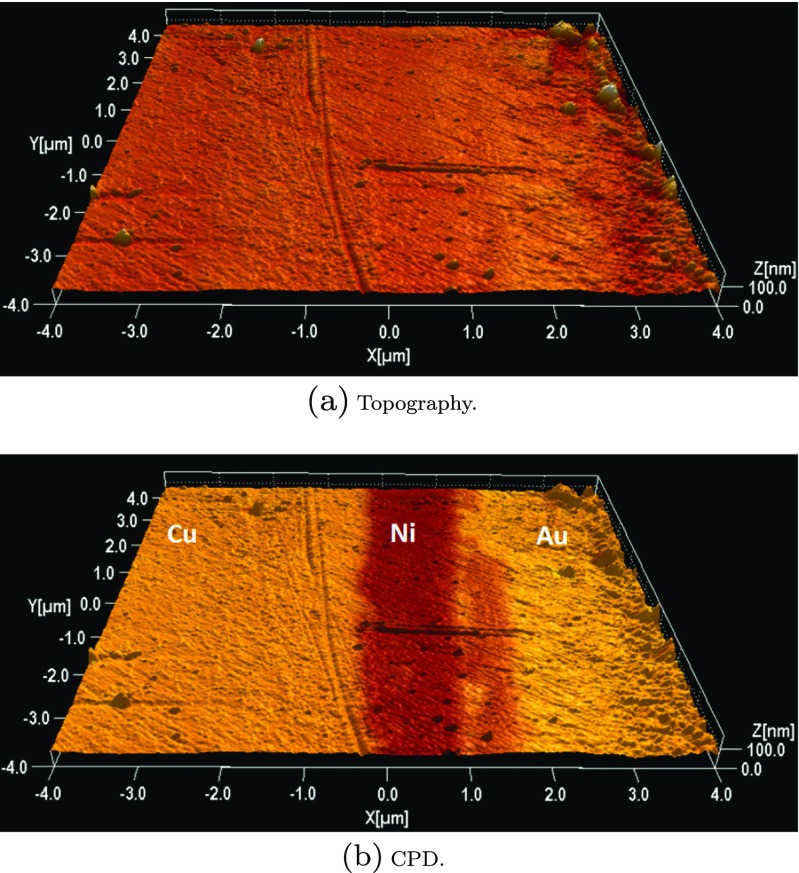
KPFM image of a reference sample consisting of an epitaxial multilayer of electroplated metals Ni and Au on a Cu substrate. Scan size: 8×8 μ*m* ^2^. The coloring in panels (**a**) and (**b**) represents topography and CPD, respectively

KPFM is a variant of atomic force microscopy (AFM) (Melitz et al. 2011) that apart from providing the conventional topographical information of an AFM system, also maps the electrical properties of the surface. This is achieved by establishing an electrical circuit between the sample and the tip, so that, the latter can sense the electrostatic forces induced by work function differences between the conducting tip and the sample, or by the presence of charges. There are several strategies for designing the feedback loop of a KPFM system, and optimize the sensitivity of such a measurement, but all of them aim at measuring the so-called contact potential difference (CPD) between the sample and the tip, which for a metal-metal interaction can be defined as

1 $$ V_{\text{cpd}} = \left( \phi_{s}-\phi_{t}\right)/e, $$

where *ϕ*_*s*_ and *ϕ*_*t*_ represent the work functions of the surface under investigation and the tip, respectively, and *e* is the electron charge. Figure 1 shows an example of results obtained with KPFM with a reference sample consisting of an epitaxial multilayer of electroplated metals nickel (Ni) and gold (Au) on a copper (Cu) substrate. As can be seen, no apparent difference between the different layers is visible in the topographical image, see Fig. 1a, while they can be clearly identified when looking at the mapped electrical properties, see Fig. 1b. These KPFM measurements and the rest of KPFM results presented in the following sections were conducted with an AFM microscope Multimode 8 (Bruker). The microscope was equipped with conductive probes of types PPP-EFM and PFQNE-AL depending on the KPFM technique used during the measurement.

### Possible artifacts in KPFM measurements

**Fig. 2:**
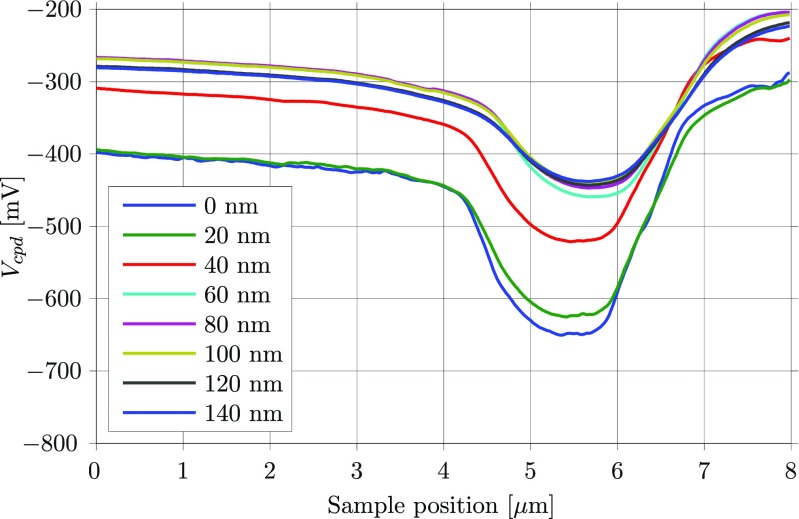
Example of AM-KPFM measurements as a function of the lift height. The curves represent the CPD potentials measured across the three metal layers (from *left* to *right*: Cu, Ni, and Au). The *x*-axis is the sample position

KPFM can provide an insightful picture of the electrical properties of a surface, but the absolute values of such an image must be treated carefully (Glatzel 2011). As presented in Eq. 1, the measured surface potentials are relative to the work function of the tip, which is a priori unknown and may change during the measurement due to wearing of the tip or changes in the atmospheric conditions. Moreover, the work function of the tip can also be influenced by parameters of the feedback loop used to sense the electrostatic forces between the tip and the sample. A clear example of this is illustrated in Fig. 2, where the cross-section of the electroplated sample presented in Fig. 1 is measured along the same line several times at different lift heights using amplitude modulation KPFM (AM-KPFM). AM-KPFM first measures the topography of the surface along a certain line and then re-scans the same line at a constant distance from the surface (typically referred to as lift height), adjusting for the measured topography, and exciting both the sample and the tip electrically in order to measure the CPD. The differences observed in Fig. 2 show that the lift height can bias the measured CPD considerably, namely, the farther away we measure from the surface, the less contrast we achieve between the different materials. This is a result of the fact that the tip interacts with a larger area of the sample when the tip is farther away from the surface, and thus, the measured CPD at a certain point is indeed the result of averaging out the CPD of a larger area (Jacobs et al. 1998; Sadewasser et al. 2009). This averaging effect is also noticeable when looking at the noise of the measured curves, as they get smoother with increasing lift height.

**Fig. 3:**
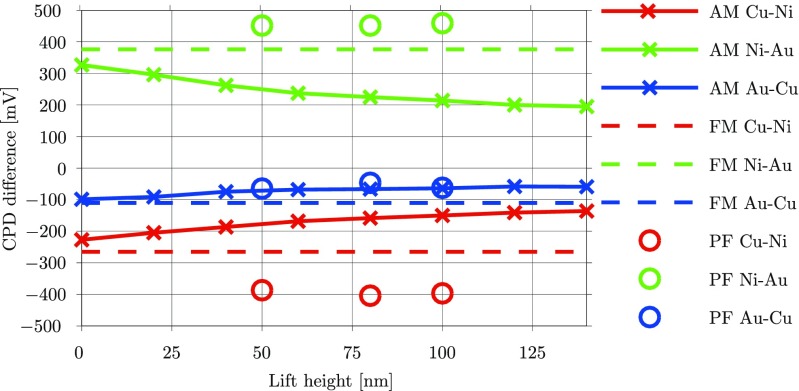
Relative comparison of KPFM techniques on the reference sample

In order to illustrate the influence of the actual KPFM technique on the measured CPD, a series of measurements on the same reference sample were carried out with three different KPFM techniques: PeakForce KPFM (PF-KPFM), frequency modulation KPFM (FM-KPFM), and AM-KPFM. The curves presented in Fig. 3 show the difference in CPD between the three metallic layers as a function of the lift height of the tip. To estimate the CPD difference across the different layers, the mean value of the surface potentials measured for each of the three materials were calculated, and subsequently pairwise subtracted from each other. This procedure was adopted because each of the KPFM techniques uses a different type of tip, which makes the comparison of absolute CPD values among the three techniques difficult. The overall results shown in Fig. 3 reveal important differences between the various techniques.

It should be noted that FM-KPFM measures topography and CPD simultaneously by driving the tip both mechanically and electrically at two different frequencies. Hence, this technique is not based on lift mode (it is rather typically referred to as single pass mode) and does not have a lift height parameter. This is the reason for displaying the FM-KPFM results as horizontal dashed lines that are independent of the lift height. Interestingly, AM-KPFM results lead to similar results as FM-KPFM when the lift height approaches 0 nm. This illustrates again that AM-KPFM measures the local properties of a surface more accurately when the tip is close to the surface. The substantial differences between AM-KPFM and FM-KPFM for larger lift heights stem from the nature of their feedback loops: while AM-KPFM reacts directly on the electrostatic forces acting on the tip, FM-KPFM detects the gradient of these forces, which makes FM-KPFM much more reactive to changes and yields a significantly higher spatial resolution (Colchero et al. 2001; Zerweck et al. 2005). By contrast, PF-KPFM seems to be considerably more independent of the lift height than AM-KPFM, in spite of the fact that it is typically implemented in lift mode. This is because PF-KPFM exploits the benefits and capabilities of PeakForce Tapping during the topographic measurement and incorporates the accuracy and higher spatial resolution of FM-KPFM during the CPD measurement (Li et al. 2013). The larger CPD differences observed between the Ni-Au layers and the Cu-Ni layers with PF-KPFM compared to the ones obtained with the other two techniques can be explained by the fact that the Ni layer was only 1 μm thick. This means that, during the scanning process, the CPD levels measured with AM-KPFM and FM-KPFM were not able to reach a stationary value on this metal of the cross-section of the electroplated sample before starting to interact with the other metals.

All in all, these results demonstrate that KPFM measurements can be influenced by the actual implementation of the technique, which points out the need for calibration procedures that make KPFM measurements comparable to other experiments.

### Example of traceability

Despite the diversity of KPFM results presented in Fig. 3, a simple calibration procedure can help to establish traceability with other experimental results. Let us consider the case where the reference sample presented in Fig. 1 is to be compared against electrochemical measurements. In this case, the electrochemical experiments are designed to measure the OCP of each metal (Cu, Ni, and Au), that is, the potential of the metal electrode relative to a reference electrode, when no external voltage or current is applied to the cell (Uosaki and Kita 1986).

In Ref. Daviðsdóttir et al. (2014), a standard three-electrode electrochemical cell setup was used for this type of measurements. There, the reference electrode used for the measurement was Hg/Hg_2_ *SO*_4_ saturated K_2_ *SO*_4_ in order to avoid any chloride contamination in the solution. The counter electrode was platinum. The electrolyte for all experiments was deionized water with analytical grade 0.1 M NaNO_3_ for increasing the conductivity of the solution. These OCP measurements were carried out by exposing a metal surface area of 9.6 cm^2^ to the solution. The volume of the electrolyte was 550 mL. The potentiostat used for the OCP experiments was from Gill AC BI-STAT. The same setup was used in this study to determine the OCP for Cu, Ni, and Au at the macroscopic scale. The mean OCP value of each metal together with the corresponding uncertainty are presented in Table 1.

**Table 1:** Experimental OCP and CPD results on Cu, Ni, and Au measured using electrochemistry and KPFM, respectively

Measurand	Cu	Ni	Au
*V*_ocp_ [mV]	312.9 ±2.4	58.9 ±0.1	358.6 ±7.6
*V*_cpd_ [mV]	189.5 ±4.4	−212.2 ±3.0	236.6 ±3.9

**Fig. 4:**
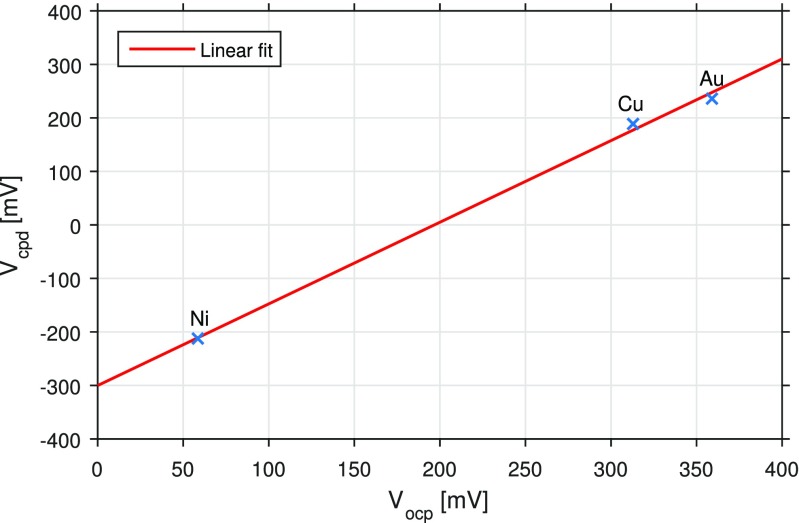
The KPFM results (*V*_cpd_) follow a linear relationship with the electrochemical results (*V*_ocp_)

In order to eliminate topographic artifacts in the KPFM measurements, the AFM was set to scan the same line on the reference sample continuously. In this way, any possible changes on the measured CPD levels are only due to changes in the measurement conditions, such as thermal drift, and not to changes in electrical properties of the sample, which could otherwise occur if measurements were conducted at different positions on the sample. Thereby, the resulting uncertainty can be strictly related to the measurement itself and not to the sample. The measurements were conducted using PF-KPFM. The mean CPD value of each metal together with the corresponding uncertainty is also presented in Table 1. When plotting the OCP potentials versus the CPD potentials, see Fig. 4, the two experiments appear to follow a linear relationship.

A simple least square procedure was used to fit the measured data to the following linear equation:

2 $$ V_{\text{cpd}} = mV_{\text{ocp}}+n,  $$

where *m* equals to 1.53 ± 0.04 (adimensional) and *n* equals to −300.2 ± 8.2 mV, when using the mean values and standard uncertainties presented in Table 1. The measurement uncertainties of the fitting parameters are here given as expanded uncertainties that are estimated according to the ‘Guide to the Expression of Uncertainty in Measurement’ (GUM) (Bureau International des Poids et Mesures 2008). A coverage factor of 2 (*k*=2) is used, which for a stochastic process that follows a Gaussian distribution corresponds to a level of confidence of 95.45 %. The fitting parameters *m* and *n* provide thereby the traceability between the two experiments. While the parameter *m* provides a scale factor between the potentials measured in the two experiments, the parameter *n* indicates the voltage offset existing between the two experiments, presumably between the reference electrode in the electrochemical experiment and the work function of the tip in the KPFM measurement.

## Assessing photocatalytic activity with KPFM

The photocatalytic properties of TiO_2_ are activated using ultraviolet (UV) light. UV photons provide enough energy to overcome the band gap of TiO_2_, and thereby, move electrons from the valence band to the conduction band. Then, the photogenerated holes and electrons (in the valence and conduction bands, respectively) diffuse to the surface, where they can oxidate and reduce oxygen and water molecules, and thereby, create reactive radicals. These radicals can degrade organic compounds on the surface of TiO_2_. All these changes in the electronic band structure affect the electrical properties of TiO_2_, and in particular, its work function. The work function is the energy necessary to bring an electron from deep inside a material (at Fermi level) to a point in vacuum immediately outside the material surface (Kahn 2016). Hence, one can interpret the changes in work function under UV radiation as a direct measurement of the photocatalytic activity of that surface. When TiO_2_ is illuminated with UV light, its work function decreases (Henning et al. 2013), meaning that it is easier to steal electrons from TiO_2_ particles and use them to generate reactive radicals such as $O^{-}_{2}$. Therefore, the larger the change in work function under UV irradiation, the more photocatalytic activity can be emanated from that surface. This effect is precisely what can be assessed by KPFM. KPFM offers the possibility of characterizing the photocatalytic activity of TiO_2_ without external agents such as methylene blue (Yan et al. 2006) or acetone by assessing simply the changes in work function, which are directly linked to the amount of electron-hole pairs generated during the activation process.

## Linking photocatalytic properties at both macro- and microscopic scales

KPFM senses the electrical properties of a surface at a microscopic scale, whereas a vast majority of photocatalytic measurements are performed at a macroscopic scale. This section presents two examples where KPFM measurements on TiO_2_-based samples are compared to different macroscopic experiments.

### Correlation between photocatalytic degradation of acetone and CPD

Four different TiO_2_ coatings (referred to as A, B, C, and D) produced by reactive magnetron sputtering were investigated. The coatings were approximately 1 μm thick and deposited onto Si substrates under different conditions. The photocatalytic activity of the coatings was investigated at the macroscopic scale by evaluating the acetone oxidation to carbon dioxide in a batch setup:

3 $$ \text{CH}_{3} \text{COCH}_{3}+4\text{O}_{2} \xrightarrow{\text{UV+TiO}_{2}} 3\text{CO}_{2}+3\text{H}_{2}\text{O}.  $$

**Fig. 5:**
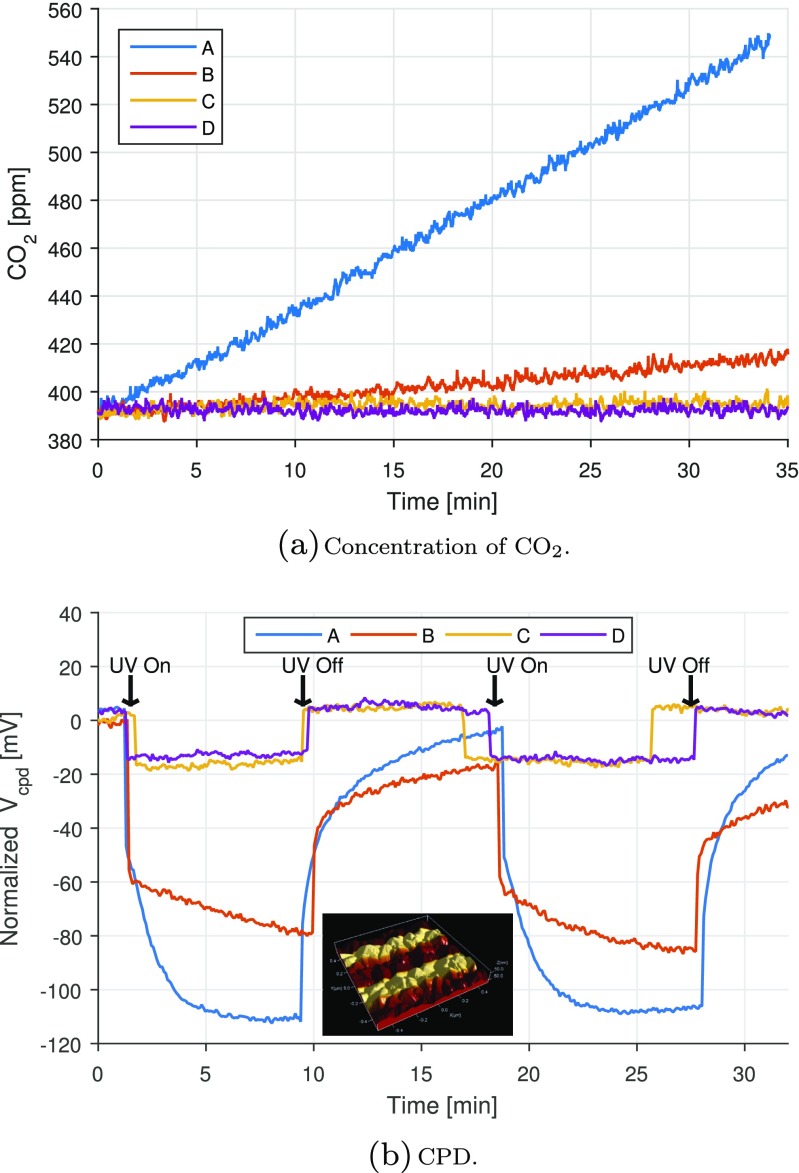
Photocatalytic activity of four different TiO_2_ coatings via (**a**) degradation of acetone and (**b**) KPFM. In (**a**), the UV light source was switched on during the entire course of the experiment. In (**b**), the inset image shows a 3D topography image of the TiO_2_ coating, where the color shading corresponds to the CPD levels measured when the UV light is turned on and off

A coated substrate was masked to an area of 17.5 cm^2^ and placed on a sample holder inside a reaction chamber containing a CO_2_ detector. The chamber was closed by a quartz lid and flushed with moisturized technical air (approximately 50 % relative humidity). The air pipes were subsequently closed, and approximately 18 μL acetone were injected into the chamber. The acetone evaporated inside the chamber, and when the CO_2_ concentration had stabilized, the sample was illuminated with 365 nm UV-A light through the quartz lid. The UV-A light intensity at the sample was approximately 6 mW/cm^2^. The setup was static, and hence, the reactants and products were not stirred inside the chamber. The photocatalytic activity was evaluated from the evolution in the CO_2_ concentration as a function of time: the larger the CO_2_ gradient, the higher the photocatalytic activity. Panel (a) in Fig. 5 shows the concentration of CO_2_ as a function of time for the four investigated coatings. Coating A is significantly more active than the rest of coatings, followed by coating B, which presents a mild response as a function of time. Coatings C and D do not seem to degrade acetone under UV exposure as the concentration of CO_2_ remains almost constant.

The macroscopic investigation of the photocatalytic activity via acetone degradation was compared with KPFM measurements. Four pieces from the original samples were cut and placed on carbon tape, so that they could be examined together in the KPFM system and measured under approximately the same conditions (similar atmospheric conditions, same tip, etc.). The samples were irradiated by a broadband light source consisting of a deuterium (UV) and a halogen (VIS) lamp from Ocean Optics. The light was guided on to the samples via a fiber. The measurements were in this case conducted using AM-KPFM. The light source was switched on and off sequentially and the resulting CPD images were processed to obtain an average CPD profile of the UV exposure, see panel (b) in Fig. 5. As can be seen, coatings A and B again present the largest responses when switching the UV light on and off. In this case, the response of coating B is closer to the one of coating A, whereas coatings C and D present a small response to UV light, which was not noticeable in the acetone degradation experiment. During a second set of KPFM measurements, the results obtained with coatings A, B, and C were well reproduced, whereas coating D had a response two and a half larger than the one presented in panel (b) in Fig. 5 (though still smaller than the responses of coatings A and B). This is probably caused by local variations of the sample surface, as KPFM is a local measurement technique.

The overall agreement between the two techniques suggests that KPFM measurements can be used for qualitative assessment of photocatalytic activity of TiO_2_ coatings. The experimental results also indicate that the correlation between the two techniques does not follow a linear relationship. The observed differences could be a result of the use of different light sources in the two experiments (narrowband vs. broadband). At the moment, the causes for explaining the differences in performance of the coatings are unknown. This is a matter of current investigations.

### Correlation between OCP and CPD experiments

**Fig. 6:**
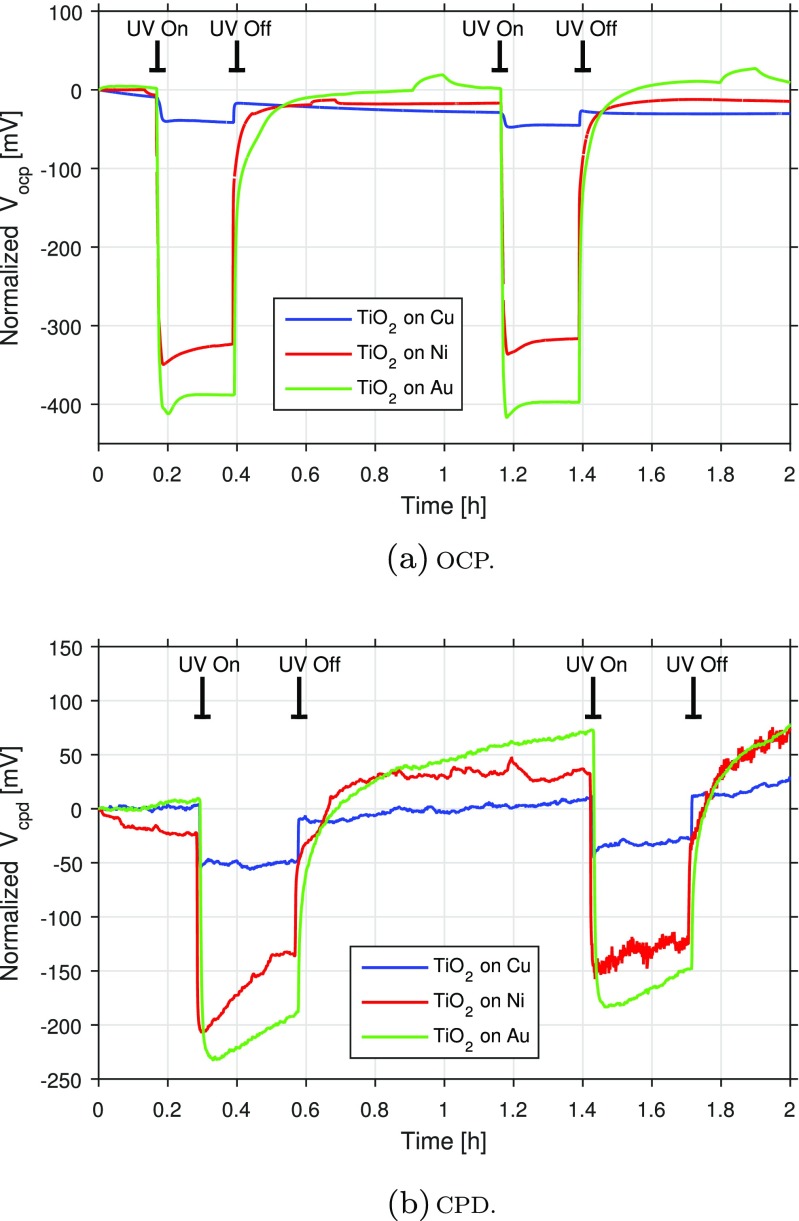
Photocatalytic activity of TiO_2_ samples at macro- and microscopic scales

In this study, anatase TiO_2_ was deposited on three different conducting substrates, namely Cu, Ni, and Au, by reactive magnetron sputtering. The thickness of the resulting coatings was approximately 850 nm for the Ni and Au substrates, whereas the TiO_2_ coating on the Cu substrate was a bit thinner, 550 nm, due to the formation of a mixture of copper oxides and titanium oxides at the coating-substrate interface (Daviðsdóttir et al. 2014). The photocatalytic performance of these three samples was examined by means of photoelectrochemical experiments that measured the OCP induced by photon excitation between two electrodes (one exposing the coating to the electrolyte). The setup was essentially the same as the one described for the electrochemical measurements in “Example of traceability”, but in this case, the coating was mounted so that it could be illuminated through the cell via a quartz window. The UV lamp was a broadband Philips home solarium, and it was placed at a distance of 35 cm from the sample. Each of the samples was exposed to UV light by switching the light source on and off sequentially. The OCPs obtained for the three samples can be seen in panel (a) in Fig. 6. The largest OCP responses under UV exposure are achieved with the Au substrate, closely followed by the Ni substrate. On the other hand, only a minor response to UV irradiation is observed for the TiO_2_ coating on the Cu substrate.

The photocatalytic activity of the same TiO_2_ coatings were also examined using AM-KPFM. The samples were irradiated by a broadband light source from Ocean Optics consisting of a deuterium (UV) and a halogen (VIS) lamp. In this experiment, the samples were also exposed to UV light sequentially, see panel (b) in Fig. 6. As can be seen, the CPD responses are in good agreement with the photoelectrochemical experiments, that is, the largest responses during UV exposure are again obtained with the Au substrate, closely followed by the Ni substrate. Similarly to the photoelectrochemical measurements, the coating on the Cu substrate exhibits a mild response compared to the other two samples.

## Conclusions

KPFM offers high resolution imaging at micro- and nanometer scale, while it simultaneously provides quantitative results regarding changes in surface potential. The possible artifacts existing in KPFM measurements have been discussed, and the differences among several KPFM techniques have been demonstrated with experimental results. A calibration procedure has been presented in order to establish traceability between KPFM measurements and a chosen macroscopic electrochemical method of sample characterization, thus providing a reference scale to compare and link the different methods. A similar approach can be adapted to other types of experiments. Furthermore, the results presented in this work show that KFPM can be used for characterizing the photocatalytic properties of TiO_2_. Changes in CPD under UV exposure are quantifiable as a direct consequence of the photogenerated electron-hole pairs that diffuse to the surface of TiO_2_, and thus, are available for photocatalytic reactions. Established measurement methods, such as acetone degradation, provide macroscopic results of the photocatalytic performance and can be compared to KPFM results. Based on this idea, the photocatalytic activity of a number of TiO_2_ samples has been examined by means of macro- and microscopic experiments. Despite the significant differences in the measurement principles, KPFM shows a good agreement with photocatalytic experiments based on electrochemistry and acetone degradation. The present work demonstrates thereby that KPFM can be linked to conventional methods operating at a macroscopic scale, while characterizing photocatalytic TiO_2_ locally, at a microscopic scale, without external agents. Therefore, KPFM can provide a valuable contribution towards the understanding, standardization and design of TiO_2_-based solutions in photocatalytic applications.
